# The influence of smartphone reduction on heart rate variability: a secondary analysis from a randomised controlled trial

**DOI:** 10.1080/21642850.2025.2546376

**Published:** 2025-08-16

**Authors:** Rachel Dale, Katja Haider, Jasminka Majdandžić, Andreas Hoenigl, Julia Schwab, Christoph Pieh

**Affiliations:** a Department for Psychosomatic Medicine and Psychotherapy, University for Continuing Education Krems, Krems an der Donau, Austria; b Faculty of Medicine and Dentistry, Danube Private University, Krems an der Donau, Austria; c Division of Oral Surgery and Orthodontics, Department of Dental Medicine and Oral Health, Medical University Graz, Graz, Austria

**Keywords:** Physiology, digital, RCT, HRV, craving, fitness tracker

## Abstract

**Background:**

Despite the benefits smartphone technology offers, our phones are available to us almost all of the time and excessive smartphone use may be linked to problematic behaviours and mental illness symptoms. Therefore management of our daily screen time is integral to wellbeing in the digital era.

**Design:**

A recent randomised controlled trial (NCT06353451) randomised university students (N = 111) to either reduce their daily phone use (intervention) or continue use as normal (control). Using a cross-over design, the control group later received the intervention. The results demonstrated that reducing smartphone use to <2hrs/day improved self-reported mental health, as compared to a control group with no change in screentime.

**Methods:**

The aim of this paper was a secondary analysis of daily heart rate variability data (HRV) measured with Fitbit devices to assess physiological changes during the intervention. A total of 45 participants provided baseline, intervention and follow-up HRV data. Mental health variables were measured using standardised questionnaires.

**Results:**

A linear multilevel regression indicated HRV significantly declined during the intervention compared to baseline. HRV during the intervention significantly correlated with craving and sleep quality. Conclusions: This may suggest that participants are experiencing a response akin to withdrawal from a behavioural addiction. Importantly, participants reported improved mental wellbeing, suggesting benefits of controlled smartphone use, but our findings provide a deeper insight into the processes underlying reduction in smartphone use and suggest craving and sleep hygiene may be important factors to additionally consider in future studies.

**Trial registration:** ClinicalTrials.gov identifier: NCT06353451.

## Introduction

Smartphones are now ubiquitous in daily life (Kemp, 2023). They offer numerous benefits for navigating the modern world, finding information and staying connected. However, emerging evidence suggests excessive smartphone use is linked to poorer mental health (Twenge et al., 2018). This implies that awareness and management of our smartphone screentime may be integral to psychological wellbeing.

In line with this, the primary analysis of the current randomised controlled trial demonstrated that reducing time on smartphones improved self-reported mental health in university students, as compared to a control group with no change in screentime (Pieh et al., 2025). This was the case for depression, wellbeing, stress and sleep quality. The subsample which strictly adhered to the screentime goal of ≤2-hours per day showed even larger effect sizes, despite no increase in physical activity being observed. This suggests a causal link between screentime and mental health. Studies investigating the effects of smartphone use on mental health predominantly use self-report measures, for example all studies included in a systematic review on effects in university students (Candussi et al., 2023). The aim of this paper was a secondary analysis using physiological data; specifically, heart rate variability. Heart rate variability (HRV), which is calculated from the R–R interval, is known to represent autonomic nervous system activity, with increased HRV reflecting increased parasympathetic nervous system (PSNS) activity and reduced sympathetic nervous system (SNS) activity. Thus, a decline in HRV is a suitable marker for stress and anxiety (Castaldo et al., 2015; Hickey et al., 2021). The HRV data provide an objectively measured complement to the self-report mental health data, offering insight into how the effects of reduced smartphone use manifest on a physiological level, thereby contributing to a more comprehensive understanding of these responses.

In the preceding study, self-reported stress declined between baseline and post-intervention and returned to baseline values at follow-up (Pieh et al., 2025). Lower HRV has also been associated with problematic internet use, suggesting the sympathetic nervous system is highly activated in excessive technology use (Cheng et al., 2023; Lin et al., 2014). Therefore we expected HRV levels to be in line with this: i.e. to increase between baseline and post-intervention as phone use is reduced, but then decrease again at follow-up. Following this we conducted exploratory analyses to investigate the psychological correlates of HRV during the intervention phase.

## Methods

### Sample and design

Details of the protocol and recruitment can be found in the registration (ClinicalTrials.gov, NCT06353451, Digital Detox Study: A Randomized Controlled Trial, 2024). University students in Austria were recruited if they were: 18–29 years, owned a smartphone and used it at least 3-hours/day, and had no diagnosed or treated mental disorder, no ongoing psychotherapy or psychopharmaceutical therapy. Moderately elevated levels in mental health questionnaires were not reasons for exclusion. Participants (N = 111) were randomised into control or intervention groups at recruitment but due to the cross-over design all participants experienced baseline, intervention and follow-up phases. Since the study phase was the key measure for the current study, all participants were combined. All participants with a minimum of one day of HRV data for each time phase (baseline (minimum 10 days), intervention (three weeks), follow-up (six weeks)) were included in the current analyses. During the intervention phase participants were asked to reduce their smartphone use to ≤2-hours/day for three consecutive weeks. Participants uploaded weekly smartphone usage screenshots, which also served to increase accountability and encourage adherence.

The ethics committee of the University for Continuing Education Krems approved the study protocol on October 23, 2023 (number: EK GZ 67/2021– 2024). Informed consent was obtained from all individual participants included in the study.

### Measures

*Heart rate variability (HRV)* was measured using Fitbit Inspire 3 watches, which participants were asked to wear daily. In order to assess overall changes in HRV over the course of the study we used RMSSD (root-mean square difference of successive R-R intervals), a time domain measure which is less affected by changes in respiration rate than frequency domain measures (Penttilä et al., 2001; Saboul et al., 2013). RMSSD is measured by Fitbit overnight to produce one value per day.

*Phase* was coded as baseline, intervention or follow-up and time was coded as day number (within each phase).

*Stress* was measured weekly with the German version of the Perceived Stress Questionnaire (PSQ) (Fliege et al., 2005; Levenstein et al., 1993). Each item is rated from almost never (1) to usually (4), with a scale rank from 0 to 100. The scale also has four subscales: worry, tension, joy, and demands. Cronbach’s alpha: overall stress scale intervention week 1 α = 0.77, intervention week 2 α = 0.78, intervention week 3 α =  0.83, tension subscale week 1 α =  0.85, week 2 α = 0.82, week 3 α = 0.81.

*Anxiety* was measured at baseline and post-intervention using the German version of the Generalised Anxiety Disorder Scale (GAD-7; Löwe et al., 2008; Spitzer et al., 2006). Items are rated on a four-point scale with a maximum score of 21. Cronbach’s alpha at week 3 α = 0.87.

*Problematic smartphone use* was measured weekly using the German version of the Smartphone Addiction Scale (Adelhardt et al., 2019; Kwon et al., 2013). To reduce burden on participants during the weekly assessments, three items from this scale were used: 5. ‘Feeling impatient and fretful when I am not holding my smartphone’, 8. ‘Constantly checking my smartphone so as not to miss updates’, 9. ‘Using my smartphone longer than I had intended’. Cronbach’s alphas for these items during the intervention were: week 1 α = 0.71, week 2 α = 0.75, week 3 α = 0.87.

*Craving* was assessed weekly with five items from the German version of the Test of Mobile Phone Dependence (TMD Brief; (Volkmer et al., 2021)). Cronbach’s alphas for these items were: week 1 α = 0.78, week 2 α = 0.88, week 3 α = 0.73.

*Sleep quality* was measured weekly with the German version of the Insomnia Severity Index (ISI), which comprises seven items on a scale from 0–4 with a maximum score of 28 (Gerber et al., 2016). Cronbach’s alphas: week 1 α = 0.82, week 2 α = 0.84, week 3 α = 0.83.

### Statistical analyses

Analyses were conducted in R version 4.1.2. Outlier RMSSD data points were defined as 3SD above or below the mean and were removed (Bourdon et al., 2018). RMSSD showed a moderate right skew and therefore the data were transformed using boxcox. A linear multilevel regression model was run with RMSSD as the outcome variable, time (day nested within phase) as the factors, without covariates, and individual as the random effect. Pairwise comparisons were run using the emmeans package, which accounts for multiple comparisons, and plots were created using ggplot2.

The subsequent analyses were explorative due to the unexpected findings of the hypothesis testing. Linear multilevel regression models were run to investigate associations between RMSSD and variables which may potentially affect physiological stress during the intervention phase only: namely, self-reported stress, the tension sub-scale of self-reported stress, anxiety, smartphone addiction, craving, and sleep quality. Our previous findings indicated that physical activity did not increase during the intervention and therefore this was not tested (Pieh et al., 2025). The threshold for statistical significance was *p* < 0.05.

## Results

**Figure 1.**
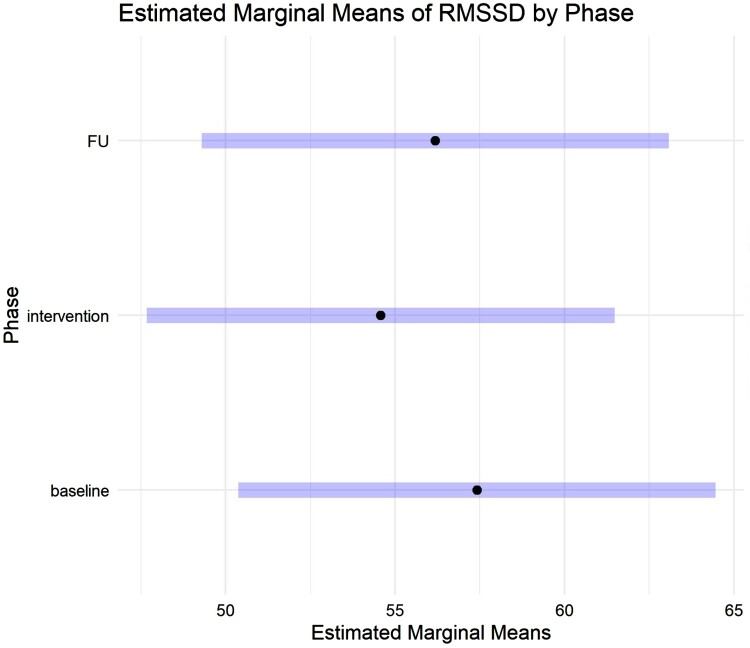
Estimated marginal mean RMSSD during each phase (baseline, intervention, follow-up [FU]).

A total of 45 participants provided HRV data for all three phases (64.4% women, mean age 23.1). There was a significant effect of phase on HRV (*F(*2) = 4.56, *p* = 0.01): HRV significantly differed between baseline and intervention (*p* = 0.02). There was no difference between baseline and follow-up (*p* = 0.51), nor intervention and follow-up (*p* = 0.12). Interestingly, HRV was lower during the intervention, reflecting higher physiological stress (Figure 1).

This is surprising considering self-reported stress decreased between baseline and post-intervention (Pieh et al., 2025) and suggests sympathetic nervous system activity, a physiological index of stress, increased during this time. Therefore we attempted to identify explanations for these seemingly contradictory results.

First we checked for an association between self-reported stress (PSQ) and HRV during the intervention period; multilevel linear models (to control for repeated measures of individual and week) showed these were not associated (*p* = 0.4). Nor was there an association with only the tension subscale of the stress measure, which may tap into more physiological aspects of stress (*p* = 0.56). Furthermore, there was no association between HRV and anxiety (GAD-7, *p* = 0.31, Table 1).

**Table 1.** Multilevel model results for associations between RMSSD and psychological variables during the intervention period.

Variable	COEFFICIENT	F-value	DF	*p*-value
Stress	−0.07	0.70	1	0.40
Tension	−0.04	0.34	1	0.56
Anxiety	−0.78	1.07	1	0.31
Smartphone addiction	−0.79	2.36	1	0.12
Craving	**−1**.**29**	**3**.**90**	**1**	**0**.**05**
Sleep	**−0**.**89**	**6**.**75**	**1**	**0**.**01**

Note: bold ≤0.05.

The PSQ & GAD-7 scales measure general stress and anxiety in daily life, but are not specifically related to smartphone use, which may be the crucial stressor here. As such we checked associations with the smartphone-related measures. There was also no association between HRV and problematic smartphone use (*p* = 0.12). However, there was an association between HRV and craving (*p* = 0.04, Table 1). Higher craving scores during the intervention were associated with lower HRV.

Additionally, there was a significant association between HRV and sleep quality, with those experiencing higher insomnia scores showing lower HRV (*p* = 0.01). Data regarding mental health outcomes can be found in Pieh et al. (2025).

## Discussion

HRV decreased during the intervention phase, where smartphone use was reduced. This suggests that while self-reported mental health improved after reducing smartphone use (Pieh et al., 2025), a sudden reduction to some extent triggers stress on a physiological level. Indeed, craving was negatively, and sleep quality was positively associated with HRV during the intervention period. Thus, while subjectively, participants felt less stressed, physiologically they may have experienced a withdrawal response similar to withdrawal from other (behavioural) addictions (Kim et al., 2018; Sun et al., 2012; Zhang et al., 2021).

So we may speculate that, while participants feel less stressed overall, their autonomic nervous system is still adjusting to reduced smartphone use, manifesting in lower HRV. Indeed, research in substance abuse has shown that HRV decreases during acute withdrawal (Chen et al., 2015; Verma et al., 2024) and craving and addiction levels are also associated with physiological measures, including HRV, in behavioural addictions (Hong et al., 2023; Kim et al., 2018). This leads to a question for future research about whether in the long-term, HRV would increase back to baseline levels as the body gets used to the new situation (of reduced smartphone use), and how this relates to subjective measures of craving over time, to gain deeper insights into HRV as a physiological marker of craving during behavioural changes. Furthermore, these results suggest that including intervention measures to manage craving may lead to reduced physiological stress and, possibly, better long-term maintenance of reduced smartphone use. Indeed HRV biofeedback interventions show promise in reducing craving in patients with substance abuse (Eddie et al., 2018; Penzlin et al., 2015). Furthermore digital mHealth interventions have been shown to improve physiological outcomes (Hayıroğlu et al., 2023) suggesting this could be done remotely via smartphones.

We also found a relationship between HRV and sleep quality during the intervention phase. Lin et al. (2014) compared those with/without insomnia and internet addiction and their findings suggest that the adverse effects of internet addiction on the autonomic nervous system function may partly result from insomnia. This suggests an interplay between behavioural addictions and sleep on HRV. Furthermore, Spiegelhalder et al. (2011) reported reduced parasympathetic activity and decreased HRV in adult subjects with objectively measured insomnia. Therefore sleep hygiene could also be a valuable addition to smartphone reduction interventions.

Some limitations should be considered when interpreting the current results. Not all participants provided HRV data and therefore the results may not be representative of the original sample. Only smartphone screen time was assessed and many people possess multiple digital devices. Although the participants were instructed not to switch to other devices, we cannot completely rule out this option. Lastly, since the sample was relatively healthy and most individuals scored at the lower end of the mental health questionnaires at baseline, a floor effect cannot be ruled out, potentially meaning that associations with HRV may not have been detected.

It is important to note that the self-reported reduction in many psychological symptoms (Pieh et al., 2025) suggests the benefits of smartphone reduction outweigh the costs. However, our findings provide a deeper insight into the processes underlying reduction in smartphone use and propose craving mitigation and sleep hygiene measures could be taken into account in future interventions to reduce the negative effects of habit change, alongside promoting the positive effects of smartphone reduction. Overall, smartphones bring numerous benefits to our lives but we must find strategies to use them in a controlled, non-excessive manner to prevent negative influences on wellbeing.

## Data Availability

The data supporting the findings of this study are available from the corresponding author upon request.

## Open Scholarship

This article has earned the Center for Open Science badges for Open Materials. The materials are openly accessible at https://osf.io/a9k76.
